# Retinal Vascular Abnormalities and Clinical Parameters in Systemic Sclerosis

**DOI:** 10.3390/jcm13102738

**Published:** 2024-05-07

**Authors:** Rosario Foti, Marco Zeppieri, Roberta Foti, Elisa Visalli, Giorgio Amato, Roberta Amato, Edoardo Dammino, Fabiana D’Esposito, Caterina Gagliano

**Affiliations:** 1 Rheumatology Unit, Policlinico San Marco Hospital, 95121 Catania, Italy; rosfoti5@gmail.com (R.F.);; 2 Department of Ophthalmology, University Hospital of Udine, 33100 Udine, Italy; 3 Eye Clinic, Catania University, San Marco Hospital, Viale Carlo Azeglio Ciampi, 95121 Catania, Italycaterina.gagliano@unikore.it (C.G.); 4 Imperial College Ophthalmic Research Group (ICORG) Unit, Imperial College, London NW1 5QH, UK; 5 Department of Medicine and Surgery, University of Enna “Kore”, Piazza dell’Università, 94100 Enna, Italy

**Keywords:** systemic sclerosis, ocular coherence tomography (OCT), ganglion cell complex thickness (GCC), nerve fiber layer thickness (RNFL), vessel density, capillary plexus

## Abstract

**Background**: Systemic sclerosis is a complex autoimmune disease characterized by vasculopathy, fibrosis, and immune dysregulation. Ocular manifestations in these patients are increasingly recognized, suggesting potential correlations between systemic vascular abnormalities and ocular microvascular changes. Advancements in molecular immunology and imaging technology using ocular coherence tomography (OCT) have unveiled intricate pathways underlying possible disease pathogenesis. Understanding the interplay between retinal vascular abnormalities and molecular immunology parameters could provide insights into disease mechanisms and potential biomarkers. **Purpose:** The aim of this study was to investigate vascular abnormalities, detected with optical coherence tomography angiography (OCT-A), in systemic sclerosis patients and to find correlations between the severity of the disease detected with molecular immunology findings and OCT-A parameters. **Methods:** A group of 32 systemic sclerosis patients were compared with 9 healthy controls. Ganglion cell complex thickness (GCC), retina thickness of the fovea and parafovea, nerve fiber layer thickness (RNFL) and cup/disc area ratio were investigated using OCT. Vessel density (VD) of the superficial (SCP) and deep capillary plexus (DCP) of the whole macular area and ETDRS grid, size of the foveal avascular zone (FAZ) and vessel density of the radial peripapillary capillary plexus (RPCP) were evaluated using OCT-A. Modified Rodnan skin score (mRSS), capillaroscopy and disease duration were used to stage disease severity. **Results:** There was a statistically significant reduction in retina thickness of the fovea and parafovea, VD of the whole DCP, VD of the SCP and DCP in ETDRS grid in the patient group compared to controls (*p* < 0.001). The patients presented a significant enlargement of the FAZ (*p* 0.005). No significant correlation between OCT and OCT-A parameters and disease severity scores was found. **Conclusions:** OCT-A could represent a non-invasive tool to detect retinal microvascular damage in systemic sclerosis.

## 1. Introduction

Systemic sclerosis (SSc) is an immune-mediated disease that has a high mortality [1]. It has a world-wide prevalence of 22 cases in 100,000, detailed in many studies with variable rates depending on country and race/ethnicity. Overall, a substantial female predominance exists, with a female-to-male ratio of 3–6:1 [2]. If, instead, a more specific focus is placed on the Italian population, in 2016, the overall crude incidence rate of systemic SSc stood at 18.5 (95% confidence interval CI 16.9–20.2) per million individuals per year. This rate was significantly higher in women, with an incidence of 31.0 (95% CI 28.1–34.1) per million, compared to men, where it was 4.3 (95% CI 3.2–5.6) per million. The overall annual prevalence was 306.1 (95% CI 301.1–311.2) cases per million population, with a notably higher prevalence in women at 530.8 (95% CI 521.5–540.2) cases per million compared to men at 67.8 (95% CI 64.4–71.3) cases per million, resulting in a female-to-male ratio of 7.8:1. The highest prevalence was observed among individuals aged 70–84 years. The crude annual mortality rate was 27.9 (95% CI 24.9–31.1) per 1000 patients [3].

The main feature of SSc is progressive fibrosis resulting from the excessive deposition of extracellular matrix components in different tissues and organs. The presence of specific autoantibodies (centromere (ACA), topoisomerase 1, or RNA polymerase III (RNApol3)), vascular damage and inflammation are also characteristic for SSc [4]. 

The activation of the adaptive and innate immune systems in systemic sclerosis (SSc) is a complex phenomenon that contributes significantly to the pathogenesis of the disease [5]. Indeed, vasculopathy is thought to be a key initiator of the heightened immune response seen in people with SSc. Vascular injury and the immune response have a complex and perhaps interdependent reciprocal interaction. The secretion of many fibrogenic substances, including transforming growth factor (TGF)-β, interleukin-13 (IL-13) and interleukin-4 (IL-4), which are significantly higher in the serum of patients with SSc, is a crucial function of immune cells. Several model systems have shown that inhibiting or depleting these components is effective in avoiding or ameliorating fibrosis. Primarily released by type 2 helper T (Th2) cells, IL-4 and IL-13 regulate the immune response by promoting the growth of B cells, the production of immunoglobulins and the expression of adhesion molecules [6]. They also enhance the synthesis of tissue inhibitors of metalloproteinase (TIMP)-1, block collagenases like metalloproteinase (MMP)1 and MMP3, and boost the production of TGFβ, fibroblast differentiation and proliferation, and extracellular matrix (ECM) [7].

One of the main players in the pathophysiology of SSc, TGFβ participates in several signaling cascades that encourage fibrogenesis and inflammation. By phosphorylating the SMAD protein, it triggers the expression of the SMAD4 gene. Additionally, it triggers the signaling pathway of mitogen-activated protein kinase (MAPK) through the pathways of c-Jun N-terminal kinase (JNK), p38 and extracellular signal-regulated kinases (ERK) 1 and 2. Through its ability to induce IL-13 production via the transcription factor GATA-3, TGFβ may have a role in the activation of immune cells and fibroblasts in a feedback loop [8].

Both innate and adaptive immunity components of the immune system have been linked to the development of SSc. Promising treatment targets might be difficult to find since it is unclear which individual cells are important for the start of the disease. SSc is characterized by the infiltration of M2 macrophages and circulating monocytes into the afflicted skin, as well as their activation. Different from inflammatory M1 macrophages, alternatively activated M2 macrophages promote tissue healing and excessive extracellular matrix deposition. M1 and M2 macrophages generate proinflammatory mediators such as IL-6 in addition to fibrogenic factors such as TGFβ, IL-4 and IL-13 [9]. 

Additionally, neutrophils contribute to the pathophysiology of SSc by releasing reactive oxygen species (ROS) that stimulate fibroblasts and generating fibrogenic cytokines as TGFβ, IL-6 and VEGF. Polymorphonuclear neutrophils from people with SSc produce more neutrophil extracellular traps (NETs) than normal. NETs are involved in immunological activation by producing interferon-α and activating plasmacytoid dendritic cells (pDCs) [10].

Dendritic cells (DCs) deliver antigens and activate naïve T cells, which modulates immunological responses and adds to the pathogenesis of SSc. Immune activation is sustained when DCs exhibit dysregulation, which is typified by enhanced expression of Toll-like receptor-8 (TLR8) on plasmacytoid DCs. This also results in heightened secretion of chemokine (C-X-C motif) ligand 4 (CXCL4) and interferon (IFN)-α.

Mast cells become early targets in the development of the illness after being drawn by local signals to fibrotic lesions. Mast cells express profibrotic substances such as TGFβ, fibronectin and platelet-derived growth factor (PDGF) in addition to histamine. Studies on mast cell depletion point to their involvement in the development of fibrotic lesions [11].

Patients with SSc have aberrant B cell homeostasis, characterized by a reduction in memory B cells and an increase in activated naïve B cells. The severity of the illness is correlated with B cell activating factor (BAFF) levels. Activated B cells secrete IL-6, which induces fibroblasts to exhibit a profibrotic state and contributes to the development of SSc. Autoantibodies, which are specific to subtypes of SSc, help in early diagnosis, prognosis, and organ involvement prediction. In SSc, there is a correlation between the severity of the illness and the dysregulation of regulatory immune cells, such as regulatory T cells (Treg) and regulatory B cells (Breg). Moreover, in SSc, natural killer (NK) cells have both pro- and anti-fibrotic actions; decreased NK cell activity and quantity may hasten the fibrosis’s advancement [12].

Systemic sclerosis affects the skin and internal organs, such as the lungs, heart, kidneys, musculoskeletal system, and the gastrointestinal tract. Skin sclerosis is a predominant symptom of SSc, which can be evaluated using the Modified Rodnan skin score (mRss) [13]. 

Microvascular damage and dysfunction are among the earliest morphological and functional indicators of systemic sclerosis. These changes and progressions can be identified through nailfold videocapillaroscopy [14].

There are two clinical subsets: diffuse cutaneous SSc (dcSSc) characterized by skin damage proximal to elbows and/or knees or affecting thorax and/or abdomen, and limited cutaneous SSc (lcSSc) characterized by skin damage distal to elbows and knees without thorax or abdomen involvement. Systemic sclerosis may lead to major disabilities due to vascular complications, cardiopulmonary involvement, inflammatory myopathy, arthritis, malnutrition from gastrointestinal tract involvement, and reduced quality of life due to psychological and social impact [15]. 

The literature discussing the ophthalmic involvement in SSc is limited, probably due to the rarity of the disease. Different eye involvement was reported in SSc: dry eyes, reduced choroidal thickness, astigmatism, posterior subcapsular cataract, increased intraocular pressure, eyelid abnormalities and retinal microcirculatory impairments [16]. Moreover, it has been shown that systemic sclerosis can often cause dry eyes due to the frequent involvement of meibomian glands, which leads to primarily lipidic lacrimal dysfunction [17]. Ocular involvement can occur at any stage of the disease, and it can also lead to irreversible damage [18].

Szucs et al. [19] categorized ophthalmic manifestations of SSc as primary, secondary or coincidental. They hypothesized that generalized vasculopathy affects the retinal and choroidal vasculature, whereas fibrosis-related mechanisms alter the ocular adnexa and the orbit. 

The aim of this cross-sectional study was to investigate the correlations between the severity of the disease and retinal vascular abnormalities detected through OCT angiography. OCT and OCT-A parameters were also compared between systemic sclerosis patients and healthy controls. According to our results, OCT and OCT-A may detect subclinical retina involvement in patients with SSc. Changes in OCT and OCT-A parameters may be early signs of microvascular impairment in these patients. OCT-A could represent a non-invasive tool to detect systemic microvascular damage in systemic sclerosis.

## 2. Materials and Methods

We included patients diagnosed with systemic sclerosis in our study. Systemic sclerosis diagnosis was established by a rheumatologist based on the diagnostic criteria outlined by the American College of Rheumatology/European League Against Rheumatism (ACR/EULAR) for SSc. This classification system consists of eight categories, with a maximum of 34 points achievable. The total score is calculated by summing the maximum weight (score) in each category. Patients scoring ≥9 points were categorized as having definitive SSc. Thirty-two patients affected by systemic sclerosis referred to the Department of Rheumatology at San Marco Hospital in Catania (Italy) were included in our study. Data obtained from the patients affected by SSc were compared with those obtained from nine age-matched and sex-matched healthy patients. In this study, the control–case ratio was 1:3.5. Patients with other systemic diseases that may impact the eye aside from SSc, such as diabetes, intraocular dense media opacities, a history of ocular trauma or surgery, concurrent intraocular diseases (including glaucoma, uveitis, retinal disorders or tumors), or ones with insufficient quality of OCT images were excluded from our study. The mean age of our sample was 48.78 years ± 12.6 SD (standard deviation), while the mean disease duration was 4.40 years ± 6.01 SD. The majority of the patients were undergoing treatment with medications such as methotrexate, iloprost, nifedipine, bosentan, rituximab and mycophenolate mofetil before and during the study. The most common comorbidities among our sample were hypertension, dyslipidemia, obesity, and chronic kidney failure.

In this study, we included systemic sclerosis patients with varying durations of disease, resulting in a heterogeneous sample ranging from relatively recent diagnoses (two years post-diagnosis) to patients in the chronic phase of the disease (more than 20 years). We then proceeded to analyze ophthalmological data and disease characteristics, ultimately correlating these parameters. This type of study, despite ocular vascular involvement not being one of the most frequent ocular complications in patients with systemic sclerosis, has proven useful for obtaining information on the prevalence of a condition or a specific characteristic within our population at a precise moment.

All enrolled patients underwent OCT and OCT angiography, using the Optovue Solix platform. The following modules were used:

- OCT Retina Cube scan to detect the ganglion cell complex (GCC) thickness (micron) and to measure retina thickness of the fovea and parafovea (micron);
- OCT-A macula 12 × 12 mm and AngioVue Retina to detect the vascular density (%) of the superficial and deep capillary plexus of the whole macular area and the ETDRS grid. It also determined the foveal avascular zone (mm^2^).
- OCT-A of optic disc 4.5 × 4.5 mm (AngioVue Disc) to detect vessel density (%) of the radial peripapillary capillary plexus
- Disc Cube to detect the Radial Nerve fiber layer thickness (micron) and cup disc area ratio.

Data from each eye of patients and controls were analyzed separately; therefore, a total of 64 affected eyes and 18 healthy eyes were studied. 

Demographic and anamnestic data were also collected for each enrolled patient. Moreover, subjects suffering from SSc were questioned about the type and duration of the disease. Nailfold capillaroscopy and Modified Rodnan skin score (mRSS) were acquired for each patient by the same expert rheumatologist. 

Nailfold capillaroscopy is usually performed by a rheumatologist using a videocapillaroscope. This examination evaluates capillary density, morphology and dimension, as well as possible micro-hemorrhages, neo-angiogenesis and avascular areas. A capillaroscopy quantitative score, based on the previously mentioned parameters, was used for staging the severity of the disease.

The Modified Rodnan skin score (mRSS) measures skin thickness in SSc; indeed, it is a summation of measurements of skin thickness in 17 various body sites including the face, upper arms, forearms, dorsum of hands, fingers, chest, abdomen, thighs, legs, and feet. The maximum score is 51 and each area is ranked as follows: 0 = normal skin, 1 = mild skin thickening, 2 = moderate skin thickening with difficulty in making skin folds and no wrinkles, 3 = severe skin thickness with inability to make skin folds between two examining fingers. 

### Statistical Data Analysis

Statistical analysis was performed using JASP software (0.16.4.0). The continuous variables were recorded and analyzed as mean ± standard deviation. The Shapiro–Wilk test was used to test the normality of the continuous variables. 

We conducted a cross-sectional study by selecting SSc patients that met our inclusion criteria and that did not meet any exclusion criteria and we matched them with healthy controls by age and sex. Moreover, all the patients underwent a complete ophthalmologic examination, as well as OCT and OCT angiography. The independent-sample *t*-test was used to compare continuous variables with normal distribution. The Wilcoxon–Mann–Whitney test was used to compare non-parametric continuous variables. Pearson’s correlation coefficient was used to measure the linear correlation between parametric oct and oct-a variables with skin score, disease duration and capillaroscopy score. Spearman’s correlation test was performed to determine linear correlation between non-parametric oct and oct-a parameters with skin score, disease duration and capillaroscopy score. The level of statistical significance was kept at *p* < 0.05. 

## 3. Results

The whole superficial vessel density was comparable between the two groups (median 48.7 in the patient group vs. 49.4 in control group, *p* 0.063). There was a statistically significant difference in the ETDRS superficial vessel density (median 48.4 in the patient group vs. 49.9 in control group, *p* < 0.01). The whole deep vessel density was significantly lower in systemic sclerosis patients than controls (median 52.5 vs. 54.35, *p* < 0.01). Patients also had lower ETDRS deep vessel density compared to healthy controls (median 52.6 vs. 54.9, *p* < 0.01). Systemic sclerosis patients presented a statistically significant enlargement of the FAZ (median 0.310 mm^2^ vs. 0.207 mm^2^, *p* 0.005). The retina thickness of the fovea was significantly reduced in the patient group (261 micron) than in controls (271 micron) (*p* 0.001). The retina thickness of the parafovea was significantly lower in the systemic sclerosis patients (mean 319.5 micron) compared to controls (mean 337.6 micron, *p* < 0.001). The radial peripapillary vessel density average was equal in the two groups (50.2 vs. 50.2). The average RNFL thickness (91.7 micron vs. 91.8 micron), GCC thickness (102 micron vs. 102.6 micron) and C/D area ratio (0.23 vs. 0.18) were comparable between the two groups (*p* 0.0777, 0.709, 0.245, respectively). 

**Figure 1:**
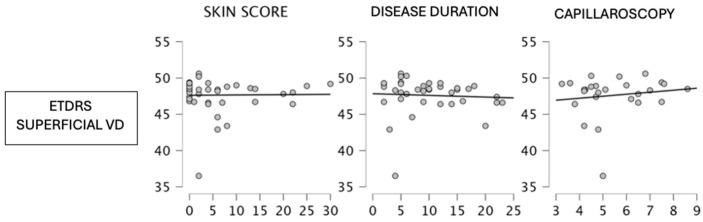
ETDRS superficial vessel density and clinical parameters correlations.

The whole superficial vessel density was negatively correlated with skin score (rho −0.144, *p* 0.382), capillaroscopy score (rho −0.016, *p* 0.938) and disease duration (rho −0.248, *p* 0.133), although the correlation was not statistically significant. The ETDRS superficial vessel density was negatively correlated with skin score (rho −0.204, *p* 0.212) and disease duration, although this was not statistically significant [Figure 1]. The whole deep vessel density was negatively correlated with skin score (rho −0.176, *p* 0.284) and disease duration (rho −0.259, *p* 0.116), although this was not statistically significant. A negative correlation between the ETDRS deep vessel density with skin score (rho −0.107, *p* 0.518) and disease duration (rho −0.215, *p* 0.196) was found. FAZ was positively correlated with the capillaroscopy score (rho 0.146, *p* 0.476), although this was not significant. The retina thickness of the fovea was negatively correlated with skin score (rho −0.117, *p* 0.477) and capillaroscopy score (rho −0.051, *p* 0.803), although this was not statistically significant. The retina thickness of the parafovea was negatively correlated with skin score (r −0.273, *p* 0.093), although this was not statistically significant. The RPC vessel density average was negatively correlated with capillaroscopy score (r −0.034, *p* 0.879) and disease duration (r −0.024, *p* 0.897), although not significantly. The radial peripapillary vessel density whole was negatively correlated with skin score (rho −0.093, *p* 0.608) and disease duration (rho −0.392, *p* 0.026). A negative correlation between average GCC thickness and capillaroscopy score was encountered (r −0.070, *p* 0.751). The average RNFL thickness was negatively correlated with disease duration (r −0.183, *p* 0.308). No significant correlation was found between the C/D area ratio with skin score, disease duration and capillaroscopy [Table 1 and Table 2].

## 4. Discussion

In patients diagnosed with systemic sclerosis (SSc), vascular manifestations often precede the onset of skin and organ fibrosis. Emerging evidence has underscored a significant pathogenic connection between early vascular injury and the subsequent development of tissue fibrosis [20].

Recent research has increasingly implicated viral infections as potential triggers that initiate vascular injury. Following this initial insult, characterized by defective vascular repair mechanisms, endothelial cells undergo activation and apoptosis. This process is accompanied by the recruitment of inflammatory and immune cells, leading to a phenomenon known as endothelial-to-mesenchymal transition. This sequential cascade instigates destructive vasculopathy within capillaries, fibroproliferative lesions in arteries and excessive fibrosis in the surrounding tissue [21]. A multitude of molecular mechanisms and pathways implicated in vascular remodeling, which subsequently leads to excessive fibrosis, have been identified. These mechanisms represent promising targets for therapeutic interventions in SSc [22,23,24].

Central to understanding the pathogenesis of SSc is the pivotal role of endothelial injury, which serves as a common link between three key features: vasculopathy, chronic inflammation and fibrosis. Exploring the processes that trigger myofibroblast differentiation in response to vascular injury will provide valuable insights into the development of targeted therapies for SSc [25].

The vascular pathogenesis of systemic sclerosis leads us to hypothesize an alteration of retinal vessels as well, since vasculopathy is the main characteristic of this disease.

Retinal microvascular abnormalities have been thoroughly investigated and documented in lifestyle-related conditions such as diabetes and arterial hypertension. These abnormalities manifest as changes in the small blood vessels within the retina, which can have significant implications for visual health and overall well-being.

Nevertheless, there has been limited exploration of retinal microvasculature in systemic sclerosis. Despite the potential relevance of understanding microvascular involvement in SSc, studies focusing on this aspect are scarce [26,27].

In our study, we found a statistically significant reduction in retinal thickness of the fovea and parafovea, suggesting that the damage to the retinal microvascular network may lead to a significant neuroretinal thinning. These findings are consistent with previous published studies [28]. 

At the same time, we showed a significant reduction in the vessel density of the whole superficial and deep capillary plexus of the ETDRS grid in the patient group compared to controls. Similar results have been described in the literature [29,30,31,32,33]. 

In a pivotal study conducted in 2019, Ranjbar et al. revealed diminished perfusion across all layers of the choroid within the submacular region among individuals afflicted with systemic sclerosis, employing OCTA technology [34].

Rommel et al. outlined findings regarding diminished perfusion in both the superficial capillary plexus (SCP) and deep capillary plexus (DCP) among individuals with systemic sclerosis (SSc). Additionally, Rommel et al. highlighted a notable reduction in macular volume (MV) in SSc patients compared to healthy individuals [35].

Mihailovic et al. demonstrated a significant reduction in the vessel density of the superficial capillary plexus and choriocapillaris in systemic sclerosis (SSc), even in asymptomatic patients [36].

Hekimsoy et al. found that the superficial capillary plexus vessel density of the whole image, fovea, parafovea and perifovea and the deep capillary plexus vessel density of the fovea were significantly lower in systemic sclerosis patients than those in healthy control subjects [32].

In our study, we also demonstrated a significant enlargement of the FAZ in systemic sclerosis, which aligns with the findings of recent studies [37].

The reduced vessel density of the retinal vascular complexes and enlargement of the foveal avascular zone may be caused by capillary dropout and vascular fibrosis, supporting the hypothesis of widespread vascular damage in systemic sclerosis. 

We also analyzed linear correlations between OCT/OCT-A parameters and clinical disease severity score (Modified Rodnan skin score, nailfold capillaroscopy parameters and disease duration). We found several correlations between these variables, suggesting that disease progression, as evaluated with clinical scores (skin score and nailfold capillaroscopy parameters), corresponds to more extensive damage to the retinal microvascular network, evaluated through OCT-A. Our results showed a statistically significant negative correlation between radial peripapillary vessel density and disease duration. Therefore, the vascular network of the optic nerve head may represent the most vulnerable site that is damaged, as systemic sclerosis progresses. To the best of our knowledge, this is the first study to provide such a finding.

Erturk et al. analyzed the differences between retinal findings in patients that exhibited Primary Raynaud’s phenomenon (PRP) only, patients with very early diffuse systemic sclerosis (VEDOSS) and patients diagnosed with systemic sclerosis (SSc). The main findings revealed significant discrepancies among different patient groups. Specifically, patients diagnosed with SSc and VEDOSS exhibited notably lower median values of superficial capillary plexus (SCP), deep capillary plexus (DCP), and optic disc vessel densities (VDs) compared to patients diagnosed with PRP. Furthermore, all measurements of SCP, DCP, and optic disc VDs displayed significant discriminatory capacity in distinguishing patients with SSc and VEDOSS from those with PRP, with the most robust discriminatory performance observed in whole and parafoveal SCP VDs [37]. These findings align with our results, indicating a statistically significant negative correlation between radial peripapillary vessel density (RPVD) and disease duration.

**Figure 2:**
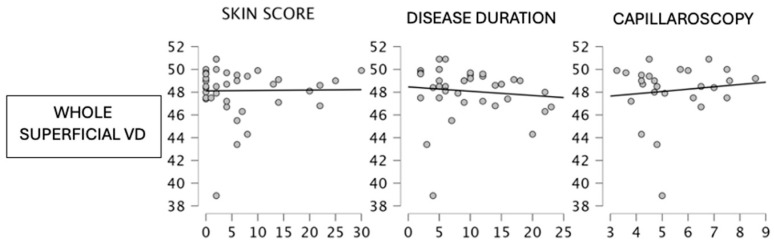
Whole superficial vessel density and clinical parameters correlations.

Unfortunately, the small sample size of our study interfered with the possibility to explore further correlations. Indeed, the remaining correlations described in our study were not significant due to a lack of statistical power. For example, whole superficial vessel density was negatively correlated with skin score (*p* 0.382) and capillaroscopy score (*p* 0.938) [Figure 2].

In 2022, Mihailovic et al. found a positive correlation between nailfold capillaroscopy and VD of the choriocapillaris and a negative correlation between skin score and VD of the superficial capillary plexus [36].

In 2020 Jakhar et al. correlated the severity of retinal changes with changes in nailfold capillaroscopy, highlighting that nailfold capillaroscopy architectural changes were more severe in patients with retinal microvascular changes such as arteriolar narrowing, arteriolar nicking, vascular tortuosity, hemorrhages, exudates, and cotton wool spots [38]. 

Detecting ocular microcirculatory abnormalities in systemic sclerosis (SSc) before systemic symptoms emerge offers a window for early diagnosis and the initiation of preventative treatments. This proactive approach could lead to timely interventions and more effective disease management.

Furthermore, the use of non-invasive techniques such as OCT and OCT angiography in patients with rheumatological conditions may serve as an additional tool for their follow-up. Indeed, the current literature contains several examples of clinical studies of this nature involving patients with rheumatoid arthritis [39,40,41], Sjogren’s syndrome [42,43,44], ANCA-vasculitis [45] and systemic lupus erythematosus [46,47], highlighting once more the significance of a multidisciplinary approach.

Conversely, this study represents the first investigation into the vulnerability of the optic nerve head’s vascular network during systemic sclerosis progression. Our findings underscore systemic sclerosis’s impact on the retinal microvascular network, particularly within delicate retinal structures. The retina’s heightened metabolic demands render it uniquely susceptible to capillary dysfunction-induced damage. Consequently, systemic sclerosis may induce vascular compromise within retinal microvasculature. Our results highlight optical coherence tomography (OCT) and OCT angiography’s (OCT-A) potential in detecting subclinical retinal involvement in systemic sclerosis (SSc) patients. Observable alterations in OCT and OCT-A parameters may serve as early indicators of microvascular dysfunction in these patients. Thus, OCT-A emerges as a promising non-invasive tool for detecting systemic microvascular damage associated with systemic sclerosis. Reductions in superficial and deep vessel density, along with FAZ enlargement, may indicate early manifestations of diffuse systemic vascular pathology in systemic sclerosis patients. Additionally, diminished fovea and parafovea thickness, as detected by structural OCT, could serve as non-invasive biomarkers for diagnosing and monitoring systemic sclerosis patients.

As previously mentioned, a considerable number of our patients had been prescribed or were currently taking medications like bosentan and iloprost to enhance their perfusion status. Considering their mechanisms of action, it could be postulated that these drugs might also exert effects on retinal vascularization. However, scant literature exists on this intriguing topic, warranting future exploration in this regard.

Some limitations of our study must be taken into account when interpreting the results. The sample size is relatively small and adequately powered studies are needed to evaluate further correlations between OCT-A parameters and clinical severity scores. This is a single-center study, limiting its generalizability. In addition, it is a cross-sectional study. Therefore, we cannot arrive at definite conclusions regarding the utility of these results in the assessment of disease progression or response to treatment. Further studies with a prospective design or from large groups with reliable longitudinal data should be performed in the future. The sensitivity, specificity and predictive values of these potential biomarkers for SSc need to be evaluated in further multi-center studies, including a larger number of patients with greater heterogeneity. 

## 5. Conclusions

Our study demonstrated that systemic sclerosis impacts the microvascular network, including the delicate structures within the retina. Given the retina’s high metabolic activity, it is particularly vulnerable to damage from capillary impairment. Consequently, systemic sclerosis can lead to vascular damage in the retinal microvascular structure. According to our results, optical coherence tomography (OCT) and OCT angiography (OCT-A) have the potential to identify subclinical retinal involvement in individuals with systemic sclerosis (SSc). Alterations observed in OCT and OCT-A parameters could serve as early indicators of microvascular impairment in these patients. Therefore, OCT-A holds promise as a non-invasive method for detecting systemic microvascular damage associated with systemic sclerosis. A reduction in the superficial and deep vessel density and enlargement of the FAZ may represent early signs of diffuse systemic vascular damage in systemic sclerosis patients. Furthermore, a reduction in the fovea and parafovea thickness, detected by structural OCT, could be used as a non-invasive biomarker for the diagnosis and follow-up of systemic sclerosis patients. It is important to recognize the different limitations of our study in order to evaluate the results. First off, given the limited sample size of this single-center study, adequately powered research is required to further assess the connections between OCT-A characteristics and clinical severity levels. Furthermore, it is impossible to draw firm conclusions about the value of these data in assessing treatment response or disease progression due to the cross-sectional nature of the study. It is also crucial to remember that the single-center setup may limit the generalizability of our findings. Prospective designs or studies with bigger cohorts and adequate longitudinal data are essential for future study. Moreover, multi-center studies with a bigger and more varied patient population are required to assess the sensitivity, specificity and predictive usefulness of putative biomarkers for SSc. In summary, our next research projects involve an extensive investigation that includes a careful analysis of the possible effects of drugs and co-occurring conditions. Furthermore, our goal is to expand the study by increasing the sample size, which will enable a stronger statistical analysis and offer better understanding of the phenomena that were seen. We will be able to clarify complex linkages and deepen our comprehension of the subject matter thanks to this extended perspective.

## Figures and Tables

**Table 1:** Descriptive statistics part 1.

	Whole Superficial VD	ETDRS Superficial VD	Whole Deep Vessel Density	ETDRS Deep Vessel Density	FAZ	Retina Thickness Fovea	Retina Thickness Parafovea
Valid	93	93	93	93	92	93	93
Missing	1	1	1	1	2	1	1
Median	48.700	48.400	52.500	52.600	0.280	261.000	320.000
Mean	47.919	47.472	50.838	51.032	0.310	257.968	319.591
Std. deviation	2.957	3.020	5.994	6.037	0.189	23.630	14.362
Shapiro-Wilk	0.753	0.734	0.579	0.530	0.745	0.940	0.977
*p*-value of Shapiro-Wilk	<0.001	<0.001	<0.001	<0.001	<0.001	<0.001	0.098
Minimum	31.300	30.800	13.700	14.400	0.000	196.000	277.000
Maximum	51.500	50.900	55.200	55.200	1.190	363.000	349.000
25th percentile	47.300	46.800	50.700	51.100	0.220	245.000	311.000
50th percentile	48.700	48.400	52.500	52.600	0.280	261.000	320.000
75th percentile	49.600	49.100	53.800	53.700	0.350	272.000	331.000

**Table 2:** Descriptive statistic part 2.

	Whole Superficial VD	RCP Vessel Density Average	RCP Vessel Density Whole	Average GCC	Average RNFL	C/D Area Ratio	Skin Score	Capillaroscopy	Disease Duration
Valid	93	82	82	82	84	80	39	26	38
Missing	1	12	12	12	10	14	55	68	56
Median	320.000	50.200	54.950	103.000	93.000	0.210	4.000	4.900	9.000
Mean	319.591	50.294	54.663	102.085	91.786	0.232	6.308	5.442	9.763
Std. deviation	14.362	2.522	2.365	7.080	12.042	0.163	7.968	1.436	6.127
Shapiro-Wilk	0.977	0.953	0.933	0.976	0.964	0.959	0.781	0.939	0.922
*p*-value of Shapiro-Wilk	0.098	0.005	<0.001	0.127	0.018	0.012	<0.001	0.126	0.012
Minimum	277.000	44.000	45.400	82.000	45.000	0.000	0.000	3.250	2.000
Maximum	349.000	60.100	58.800	115.000	118.000	0.730	30.000	8.600	23.000
25th percentile	311.000	49.200	53.325	98.000	85.000	0.117	0.000	4.313	5.000
50th percentile	320.000	50.200	54.950	103.000	93.000	0.210	4.000	4.900	9.000
75th percentile	331.000	51.625	56.200	106.750	100.000	0.333	8.000	6.500	14.000

## Data Availability

Data are contained within the article.
